# Improved Phylogenetic Analyses Corroborate a Plausible Position of *Martialis heureka* in the Ant Tree of Life

**DOI:** 10.1371/journal.pone.0021031

**Published:** 2011-06-24

**Authors:** Patrick Kück, Francisco Hita Garcia, Bernhard Misof, Karen Meusemann

**Affiliations:** 1 Zoologisches Forschungsmuseum Alexander Koenig (ZFMK), Bonn, Germany; 2 Department of Entomology, California Academy of Sciences, San Francisco, California, United States of America; Natural History Museum of Denmark, Denmark

## Abstract

Martialinae are pale, eyeless and probably hypogaeic predatory ants. Morphological character sets suggest a close relationship to the ant subfamily Leptanillinae. Recent analyses based on molecular sequence data suggest that Martialinae are the sister group to all extant ants. However, by comparing molecular studies and different reconstruction methods, the position of Martialinae remains ambiguous. While this sister group relationship was well supported by Bayesian partitioned analyses, Maximum Likelihood approaches could not unequivocally resolve the position of Martialinae. By re-analysing a previous published molecular data set, we show that the Maximum Likelihood approach is highly appropriate to resolve deep ant relationships, especially between Leptanillinae, Martialinae and the remaining ant subfamilies. Based on improved alignments, alignment masking, and tree reconstructions with a sufficient number of bootstrap replicates, our results strongly reject a placement of Martialinae at the first split within the ant tree of life. Instead, we suggest that Leptanillinae are a sister group to all other extant ant subfamilies, whereas Martialinae branch off as a second lineage. This assumption is backed by approximately unbiased (AU) tests, additional Bayesian analyses and split networks. Our results demonstrate clear effects of improved alignment approaches, alignment masking and data partitioning. We hope that our study illustrates the importance of thorough, comprehensible phylogenetic analyses using the example of ant relationships.

## Introduction

Recently, a spectacular and rare new subfamily of ants was described from the Brazilian Amazon with new implications for the ant tree of life. The monotypic subfamily, Martialinae was characterized by a single worker that shows remarkable morphological features [1]. It is a small, blind, pale, and most likely hypogaeic predator that lives either in the leaf-litter stratum or directly within the soil. Some morphological characters, such as the absence of eyes and frontal lobes, fully exposed antennal sockets, and a flexible promesonotal suture, indicate a closer relationship to the also small, eyeless, subterranean, and predatory ant subfamily, Leptanillinae [2]. Other characters, like a strongly reduced clypeus and long forceps-like mandibles, justify the establishment of a taxon Martialinae [1]. More important, this new subfamily was presented as a putative sister group to all other extant ants on the basis of the molecular analyses of three nuclear genes, the small and large nuclear subunits 18S and 28S rRNA and elongation factor EF1aF2 [1]. Previous molecular studies had proposed the subfamily Leptanillinae as a sister group of all other extant ants [3]–[5]. The proposed sister group relationship of leptanillines suggested in these studies, as well as the one presented for Martialinae by Rabeling et al. (2008) [1], is of high significance for a better understanding of ant relationships and ground plan characters. These results strongly support the scenario of a small, eyeless, and hypogaeic predator as an ancestor of modern ants [1], [3], [4], but contradict previous morphological studies, which assumed that ancestral ants were larger, more wasp-like, epigaeic foragers with well-developed eyes [6]–[9]. Therefore, the phylogenetic position of Martialinae and Leptanillinae within the ant tree of life still awaits a clear resolution.

Rabeling et al. (2008) [1] presented a Bayesian tree with resolved single inter- and intra subfamily relationships and proposed Martialinae as the earliest branch (posterior probability 0.91) within the ant tree of life. Recent studies have shown that Bayesian analyses tend to overestimate the potential signal within data and provide high support values, even if the data is completely uninformative [10], [11]. Furthermore, Bayesian approaches show a much higher type I error rate (the possibility that erroneous conclusions will be drawn more often), especially in the case of model misspecification [11]. Bayesian posterior probability values are substantially higher than corresponding bootstrap values [10]–[13]. Suzuki, Glazko & Nei [10] showed in simulation studies that Bayesian support values “can be excessively liberal when concatenated gene sequences are used”. Bootstrap values are in general more conservative and more reliable in assessing the robustness of phylogenetic trees which should be preferable in phylogenetic analyses [10], [11], [13]. Therefore, we suggest that topologies inferred with Maximum Likelihood (ML) analyses in combination with a sufficient number of bootstrap replicates provide a more realistic picture of the underlying signal.

We re-analysed the data of Rabeling et al. (2008) [1] using partitioned and unpartitioned ML approaches with a sufficient number of bootstrap replicates. Despite the mentioned criticisms on Bayesian analyses, we additionally conducted comparable Bayesian analyses to see whether any of our Bayesian topologies support the relationships found by Rabeling et al. (2008) [1], especially with respect to deep splits. For alignment masking we applied the software ALISCORE. Recent studies have shown that alignment masking of positions that can not be aligned unambiguously is strongly recommended to improve the signal-to-noise ratio in multiple sequence alignments prior to tree reconstruction. Several automated software tools have been developed [14]–[18] that offer a more comprehensible alignment masking than a manual exclusion of sites. ALISCORE is a parametric masking approach that identifies randomised alignment sections by using a Monte Carlo resampling within a sliding window [17], [18]. The approach assumes that the score of inaccurate and ambiguous alignment sections will not be distinguishable from randomly similar aligned sequences. Therefore, ALISCORE compares the score of originally aligned sequences with scores of randomly drawn sequences of similar character composition. ALISCORE has been successfully tested both in simulations [17] and on real data sets [18], and has been used in recent molecular phylogenetic studies [19]–[23].

## Results

### Alignment masking, number of bootstrap replicates and likelihood scores

Alignment masking remarkably improved data structure, which is visualised by comparing split networks derived from the unmasked and masked alignments. The split (NeighborNet) network [24]–[26] from the masked alignment obviously showed less conflict than the split network from the unmasked alignment, especially within subfamilies of formicoids. Nevertheless, conflicting signal is obvious, e.g. within poneroids or dorylomorphs (see Figure S1).

We determined the number of sufficient bootstrap replicates for our ML analyses using the ‘bootstopping criterion’ according to Pattengale et al. (2010) [27] (see method section). Our unmasked data set converged after 2,400 bootstrap replicates, our masked-unpartitioned data set after 3,400 bootstrap replicates, and the masked-partitioned data set after 4,100 bootstrap replicates applying the Weighted Robinson-Foulds (WRF) distance criterion [27] with an extended majority-rule (MRE) consensus tree criterion and a cutoff value of 0.01. Thus, the number of 5,000 bootstrap replicates chosen for our ML analyses had been sufficient for all of our data sets.

Our partitioned ML analysis of the masked data set clearly outperformed the masked-unpartitioned data set in terms of likelihood scores (masked-partitioned: *ln* = −49230.716; masked-unpartitioned: *ln* = −52002.229).

### Phylogenetic relationships

#### Placement of Leptanillinae and Martialinae

**Figure 1:**
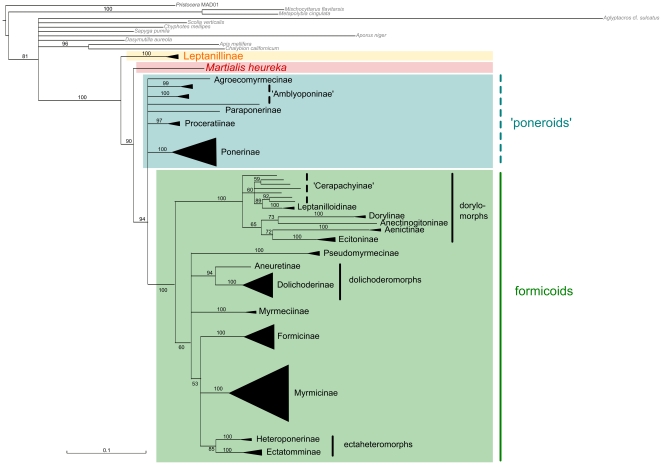
ML topology inferred from the unmasked, unpartitioned data set. Schematised ML topology with branch lengths inferred from the unmasked supermatrix (best ML tree, majority rule, 5,000 bootstrap replicates). Quotation marks indicate non-monophyly.

**Figure 2:**
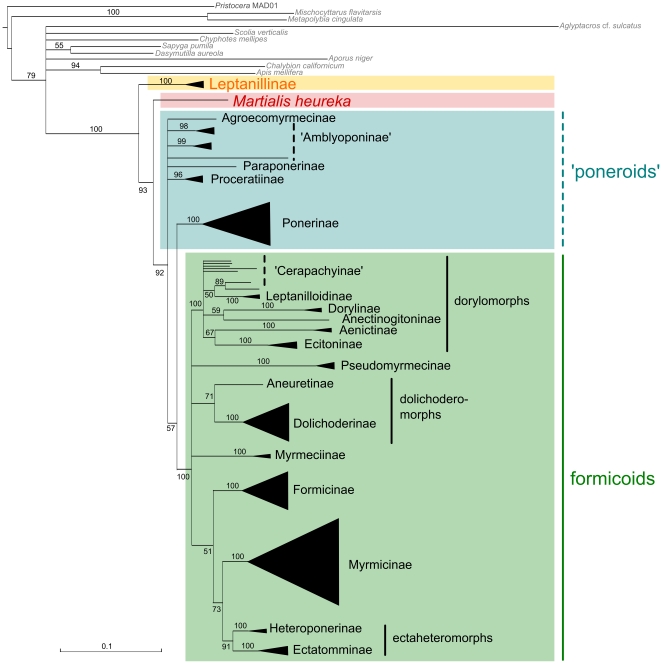
ML topology inferred from the masked-unpartitioned data set. Schematised ML topologies with branch lengths inferred from the masked supermatrix. Best ML tree of the masked-unpartitioned analysis (739 positions excluded from the unmasked alignment), majority rule, 5,000 bootstrap replicates. Quotation marks indicate non-monophyly.

**Figure 3:**
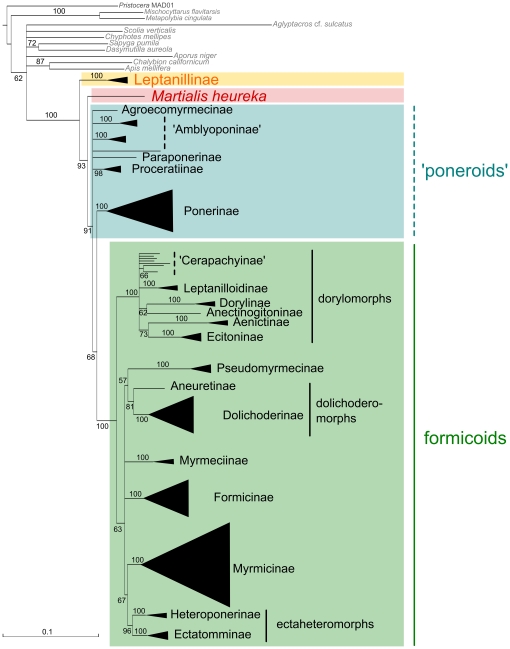
ML topology inferred from the masked-partitioned data set. Schematised ML topologies with branch lengths inferred from the masked supermatrix. Best ML tree, of the masked-partitioned analysis (739 positions excluded from the unmasked alignment+one bp to correct the reading frame), majority rule, 5,000 bootstrap replicates. Quotation marks indicate non-monophyly.

All ML and Bayesian topologies suggested a clade including Leptanillinae + all remaining ant subfamilies with maximum support (Figures 1, 2, 3, Table 1, and Figures S2, S3, S4, S5, S6, S7). Martialinae always split off as a second branch and form a clade with poneroids and monophyletic formicoids. Applying an approximately unbiased test (AU test) [28] for all ML topologies, the Null hypothesis (H_0_) assumes that either Leptanillinae as a sister group of remaining Formicidae and Martialinae as second branch in the ant tree of life or vice versa, are not significantly different. While H_0_ was not significantly rejected for our unmasked data set (*p* = 0.120), both ML topologies of our masked data sets significantly outperformed H_0_. Both AU tests of the masked and the masked-partitioned data set significantly rejected H_0_ (masked: *p*<0.0001; masked-partitioned: *p* = 0.046). Leptanillinae as the first split within the ant tree of life was also supported by our split network analyses. Both split networks (masked and unmasked) showed less conflict for Leptanillinae as the first split than for Martialinae (see Figure S1).

**Table 1:** Selected clades with posterior probability and bootstrap support values.

	Bayes posterior probabilities [bpp]			ML bootstrap support [bs]		
	unmasked	masked	masked-part.	unmasked	masked	masked-part.
Clade 1	1	1	1	100	100	100
Clade 2	1	1	1	90	93	93
poneroids	0.86	–	–	–	–	–
Amblyoponinae	–	–	0.77	–	–	–
(Ponerinae,formicoids)	–	0.97	1	–	57	68
formicoids	1	1	1	100	100	100
dorylomorphs	1	1	1	100	100	100

Selected clades with bayesian posterior probability [bpp] and bootstrap support [bs] values recovered in our Bayesian (Bayes) and Maximum Likelihood (ML) topologies. Clade 1 (Leptanillinae,(Martialinae, remaining ants)) and (Martialinae(poneroid/formicoid clade)) are resolved in all Bayesian and ML topologies. Poneroids are not monophyletic with the exception of the unmasked, Bayesian topology (weakly supported). Amblyoponinae are only monophyletic within the Bayesian masked-partitioned topology. A clade (Ponerinae,formicoids) with a subsequent paraphyly of poneroids, is suggested by all masked topologies with high Bayesian posterior probability (bpp) but low bootstrap (bs) support. Dorylomorphs are monophyletic with exception of the masked-unpartitioned ML topology.

#### Relationships of poneroids and formicoids

None of our topologies recovered a clade poneroids, except the Bayesian topology derived from the unmasked data set (0.86 bpp, see Figure S4). Further, all ML and Bayesian topologies failed to resolve the relationships between Agroecomyrmecinae, Amblyoponinae, Paraponerinae, and Proceratiinae. Conflicting signal among these subfamilies is seen in both split networks, but the masked network shows less conflict (Figure S2b). In contrast to our unmasked data, all masked approaches resolved a (Ponerinae, formicoids) clade with weak bootstrap and high Bayesian support values (masked-unpartitioned: 57% bs, 0.97 bpp; masked-partitioned: 68% bs, 1 bpp; Figures 2 and 3, Table 1, and Figures S6 and S7). A formicoid clade was maximally supported in all topologies (100% bs, 1 bpp).

Within formicoids, a dorylomorph clade was recovered in all our trees (100% bs, 1 bpp; Figures 1, 2, 3, Table 1 and Figures S2, S3, S4, S5, S6, S7). Four of six topologies suggested a clade dorylomorphs+formicoids. However, in the ML masked-unpartitioned topology, the placement of dorylomorphs remained unresolved. In the unmasked Bayesian topology, a clade dorylomorphs+Pseudomyrmecinae was present, but with weak support (see Figure S4). Concerning the relationships between dolichoderomorphs, Myrmeciinae, and Pseudomyrmecinae, we did not obtain an unequivocal resolution from any topology. The relationships between Formicinae, Myrmicinae and ectaheteromorphs were not resolved by our ML topology of the unmasked data set, whereas the trees of both masked approaches showed weak node support for a clade Myrmicinae+ectaheteromorphs (unpartitioned: 73% bs; partitioned: 67% bs). This clade was also resolved in all Bayesian topologies with moderate support (see Figures S5, S6, S7).

## Discussion

A clade Leptanillinae + all remaining ant subfamilies is highly supported in all our ML and Bayesian analyses. This result is significant with AU tests for the masked-unpartitioned and masked-partitioned approach. Our split network analyses similarly corroborate this scenario. This is also congruent to earlier molecular studies [3], [4], but contradicts the results of Rabeling et al. [1]. Based on our re-analyses of the respective data set [1] and other molecular studies [3]–[5], [29], [30], we suggest that, at present, it seems unlikely that Martialinae are the sister group to all other recent ant subfamilies.

The placement of Martialinae suggested by Rabeling et al. [1] could be due to inferior sequence alignments or confounding effects of randomized alignment sections. The MAFFT-L-*ins-i* algorithm applied in our study was shown to be one of the most accurate available alignment algorithms, and can be considered to be the best choice for sequence alignments [31], [32]. Still, 739 alignment positions were identified by ALISCORE as potentially randomised and therefore excluded. ALISCORE and subsequent alignment masking increased the signal-to-noise ratio within the data, but influenced our tree topologies only marginally. However, a positive effect of the masking approach is clearly shown by a strong decrease of contradictory signal within the masked alignment, especially for deeper splits (Figure S1). Partitioning of the masked data set leads to an increased likelihood score, and higher node resolution within formicoids. Martialinae are again resolved as the second branch (cf. Figures 1 2, 3, Table 1, and Figures S2, S3, S4, S5, S6, S7) avoiding possible artifacts due to noise.

Discrepancies between our results and the results of Rabeling et al. [1] could further be explained by an insufficient number of boostrap replicates (ML approach) and an insufficient number of Bayesian generations. They conducted 500 bootstrap replicates for the ML approach [1] *versus* 5,000 bootstrap replicates in our study. Pattengale et al. (2010) [27] showed in a recent study on ‘bootstopping’ that the number of bootstrap replicates for accurate confidence values is strongly dependent on the data set. In testing the performance and accuracy of bootstrap criteria on real DNA alignments, they showed that a range of 100 – 500 bootstrap replicates is usually sufficient. Still, in some cases a much higher number of up to 1,200 replicates was necessary to deliver support values that are equally robust as those in the reference tree with 10,000 replicates. Most differences between reference and ‘bootstopped’ topologies occurred on poorly supported branches (<75% bs). Since the bootstrap support in the ML tree of Rabeling et al. [1] for a clade Martialinae+remaining ants is only 76.2%, 500 replicates might have been insufficient. In contrast, our support values derived from 5,000 bootstrap replicates are evaluated and confirmed by *a posteriori* ‘bootstop tests’ (see results). As mentioned above, single data sets of earlier studies [3], [4] propose Leptanillinae as a sister lineage to all other ants. However, it should be considered that the subfamily Martialinae was just discovered in 2008. Therefore, Moreau (2009) [29] combined data sets of Brady et al. (2006) [3], Moreau et al. (2006) [4], and Rabeling et al. (2008) [1] to a supermatrix in which the relationship of Leptanillinae and Martialinae was unresolved.

Our analyses showed that an exclusion of randomised sections improved the resolution between Ponerinae and the formicoids (Figure 2, 3 and Figures S1, S3, S4, S6, S7). Alignment masking led to a placement of Ponerinae next to formicoids (Table 1). Discrepancies between low bs and high bpp support values seem to confirm typical observations considering Bayesian analyses [10]–[13]. The relationships between the Amblyoponinae, Agroecomyrmecinae, Paraponerinae, and Proceratiinae remain unresolved in most of our topologies. Only the Bayesian topology of the masked-partitioned data set show monophyletic Amblyoponinae with weak support (Tab. 1). Thereby, Amblyoponinae branch off as a third split (0.84 bpp) within the ant tree of life. The monophyly of Amblyoponinae has been favoured by earlier studies [3]–[5], [29]. Therefore, we conclude that more genes are necessary to robustly resolve an amblyoponine clade as well as relationships between Amblyoponinae, Agroecomyrmecinae, Paraponerinae, and Proceratiinae. All our topologies highly support a dorylomorph clade. Our unmasked and masked-partitioned topology and both Bayesian topologies derived from our masked approaches corroborate a placement of the dorylomorphs next to the remaining formicoids. This hypothesis stands in concordance with other studies [1], [3], [4]. Finally, the non-monophyly of cerapachyines within the dorylomorphs is consistent with these studies.

Compared with Brady et al. 2006 [3], the inclusion of Martialinae reduce the branch lengths for leptanillines and formicids, although the branch separating ants from the aculeate outgroup Hymenoptera still remains relatively long. However, with current methods and the available data, it is not possible to assess putative long branch artifacts like discussed in Brady et al. 2006 [3]. It is possible that new molecular sequence data might ‘improve’ the current ant tree of life. It is possible that a data set with most signal coming from rRNA genes might not be sufficient to support a robust ant tree (cf. Figure S1). For a deeper insight into subfamily relationships, multi-gene analyses of genomic/EST data and a more exhaustive taxon sampling combined with improved phylogenetic approaches seem indispensable.

## Materials and Methods

### Data set

We used molecular data previously published by Rabeling et al. (2008) [1]. In accordance to [1], we used the data matrix of Brady et al.(2006) [3] kindly provided by S. Brady. We added respective sequences of *Martialis heureka* [1] from GenBank (http://www.ncbi.nlm.nih.gov/). The data set comprised three genes of 152 taxa subdivided into 21 ant subfamilies and 11 outgroup taxa. Sequence data included elongation factor 1-alpha F2 (EF1aF2, nuclear protein coding gene), 18S rRNA and 28S rRNA (nuclear ribosomal genes).

### Alignment

Single genes were aligned separately using the local L-ins-i algorithm of MAFFT version 6.717 [33]. The L-*ins-i* algorithm is an iterative progressive algorithm which outperformed other methods in benchmark tests [31], [32]. Each of the three sequence alignments (18S, 28S, and EF1aF2) was screened for randomised sections with ALISCORE [17] using all possible pairwise comparisons and a window size w = 6. Within ALISCORE, gaps were treated as ambiguous characters. Randomised sections (28S rRNA: 725 base positions (bp); 18S rRNA: 14 bp) were excluded with ALICUT [34]. In the EF1aF2 alignment, no randomised positions were detected. Single genes were concatenated using FASconCAT version 1.0 [35]. The concatenated supermatrix of the masked approach included 4,315 characters while the unmasked supermatrix comprised 5,054 characters. All alignments (fasta format) and the respective character partitions are provided in Information S1, S2, S3, S4 and are freely available from http://www.zfmk.de.

### Phylogenetic reconstructions

#### Split networks

We computed NeighbourNetworks [24]–[26] with SplitsTree 4.10 [25] to visualise the data structure of the unmasked and masked alignments. NeighborNetworks were calculated applying uncorrected p-distances for the unmasked alignment and the masked alignment used for the masked-partitioned analyses. NeighborNetwork graphs give an indication of noise, signal-like patterns and conflicts within a multiple sequence alignments.

#### Maximum Likelihood Analyses

We estimated a Maximum Likelihood (ML) topology for the unmasked supermatrix and the masked supermatrix in non-partitioned analyses with RAxML [36] using RAxMLHPC-PTHREADS [37], version 7.2.6. A third topology was reconstructed from the masked supermatrix with four partitions according to the setup described for the Bayesian analyses in Rabeling et al. (2008) [1] with the RAxMLHPC-HYBRID [38], version 7.2.6. The first partition included the 18S, the second partition the 28S. The third partition comprised the 1st and 2nd codon position of EF1aF2, the fourth partition included the 3rd codon position of EF1aF2. We identified the correct reading frame and excluded the first position of the EF1aF2-alignment. Therefore, the EF1aF2-alignment was 1 bp shorter (516 bp) than that described in Rabeling et al. (2008) [1].

We conducted rapid bootstrap analyses and a thorough search for the best ML tree using GTR+α with 5,000 bootstrap replicates. We evaluated the number of necessary bootstrap replicates *a posteriori* for each data set according to the bootstop criteria based on the Weighted Robinson-Foulds (WRF) distance criterion [27] using RAxML 7.2.6 for the extended majority-rule (MRE) consensus tree criterion. We chose a cutoff value of 0.01 to ensure a sufficient number of bootstrap replicates. In final trees, clades with a bootstrap support (bs) below 50% were considered unresolved. All analyses were performed on HPC LINUX clusters of the ZFMK, Bonn, Germany. Trees were edited with the software TreeGraph 2 [39].

To test alternative placements of Martialinae and Leptanillinae as suggested by Rabeling et al. (2008) [1], we exchanged the position of Martialinae and Leptanillinae in our best trees (unmasked, masked-unpartitioned and masked-partitioned). We compared alternative tree topologies by performing an AU test [28] for each data set. Therefore, we optimised branch lengths for alternative topologies. Subsequently, we calculated per site log Likelihood scores using RAxML 7.2.6. AU tests were performed with CONSEL [40], version v0.1i.

#### Bayesian Analyses

Bayesian phylogenies were calculated using MrBayes [41], [42] for three data sets also used in our ML analyses. Topologies were inferred from (i) the unmasked superalignment (ii) the masked superalignment, non-partitioned and (iii) the masked superalignment with four partitions according to [1] and our ML analyses. Similar to Rabeling et al., we used MrBayes v3.2 (an unreleased version of MrBayes; the source code was downloaded from the current version system in January, 2011). Convergence of parameters of the Bayesian analyses was assessed with the software Tracer v1.5 [43].

We chose the sequence evolution model GTR+*Γ* for all three data sets (i) – (iii) for accuracy of comparison with our ML analyses. Parameters of the model (i.e., base frequencies, transition/transversion ratio, and rate variation shape parameter) were unlinked across partitions. According to Rabeling et al., Metropolis coupling was used with eight chains per analysis and a temperature increment of 0.05 [1]. For analysis (i) and (ii) we ran 30 million generations with a sample frequency of 200. For analysis (iii) we ran 28,130,500 generations with a sample frequency of 100. After checking all analyses for parameter convergence in Tracer v1.5, we discarded a burn-in of 10% for each analysis. After discarding the burn-in, majority rule consensus trees with posterior probabilities were calculated from all sampled trees within MrBayes. All analyses were performed on HPC LINUX clusters of the ZFMK, Bonn, Germany. Trees were edited with the software TreeGraph 2 [39].

## Supporting Information

Figure S1 **NeighborNet graphs with uncorrected p distances inferred with Splitstree version 4.10 from the unmasked and masked alignment.**
(PDF)

Figure S2 **RAxML-phylogram (majority rule) inferred from the unmasked alignment.**
(PDF)

Figure S3 **RAxML-phylogram (majority rule) inferred from the masked-unpartitioned approach.**
(PDF)

Figure S4 **RAxML-phylogram (majority rule) inferred from the masked-partitioned approach. (refer to** **Figure 2** **and** **3** **in the manuscript).**
(PDF)

Figure S5 **Bayesian-phylogram (majority rule consensus tree) inferred from the unmasked alignment (28,130,500 generations, samplefrequency 100, burn-in: 10% discarded).**
(PDF)

Figure S6 **Bayesian-phylogram (majority rule consensus tree) inferred from the masked-unpartitioned approach (30 million generations, sample frequency 200, burn-in: 10% discarded).**
(PDF)

Figure S7 **Bayesian-phylogram (majority rule consensus tree) inferred from the masked-partitioned approach. (30 million generations, sample frequency 200, burn-in: 10% discarded).**
(PDF)

Information S1 **Unmasked alignment in fasta format.**
(PHY)

Information S2 **Masked alignment in fasta format used for the masked-unpartitioned analyses.**
(PHY)

Information S3 **Masked alignment in fasta format used for the masked-partitioned analyses.**
(PHY)

Information S4 **Character partition file (plain text format) for the masked alignment used for the masked-partitioned analyses.**
(PHY)
