# Supplementary material for: Improved Phylogenetic Analyses Corroborate a Plausible Position of Martialis heureka in the Ant Tree of Life
Source: PLoS One. 2011 Jun 24;6(6):e21031. doi: 10.1371/journal.pone.0021031 (PMC3123331; doi:10.1371/journal.pone.0021031)
Supplement: Figure S1 — NeighborNet graphs with uncorrected p distances inferred with Splitstree version 4.10 from the unmasked and masked alignment. (PDF) [file pone.0021031.s001.pdf]

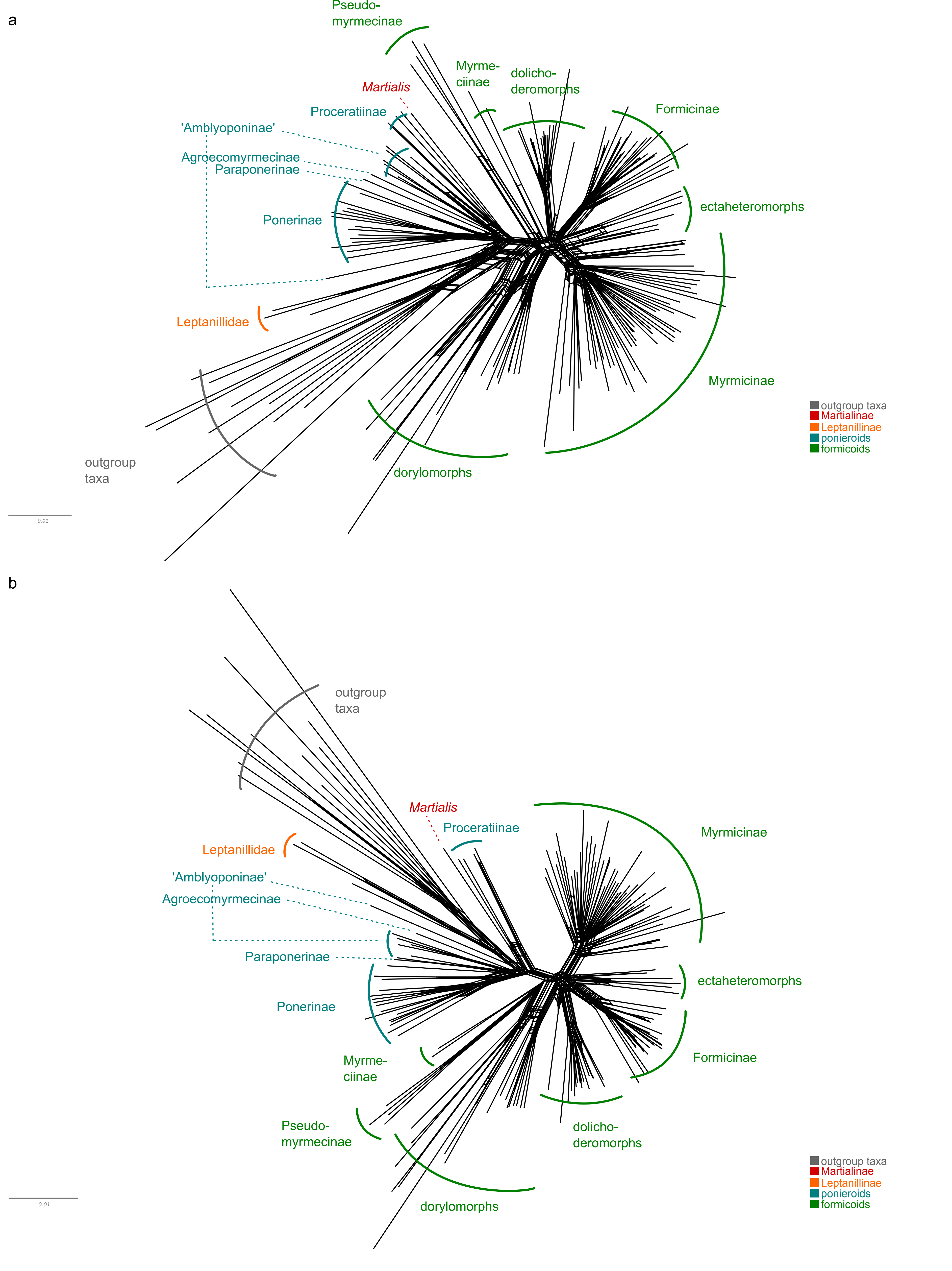

Figure S1: Neighbornet graphs with uncorrected p distances inferred with Splitstree version 4.10. **a:** Split network based on the unmasked alignment**. b:** Split network based on the masked alignment which was used for the masked-partitioned analyses.
