# Supplementary material for: Improved Phylogenetic Analyses Corroborate a Plausible Position of Martialis heureka in the Ant Tree of Life
Source: PLoS One. 2011 Jun 24;6(6):e21031. doi: 10.1371/journal.pone.0021031 (PMC3123331; doi:10.1371/journal.pone.0021031)
Supplement: Figure S4 — RAxML-phylogram (majority rule) inferred from the masked-partitioned approach. (refer to Figure 2 and 3 in the manuscript). (PDF) [file pone.0021031.s004.pdf]

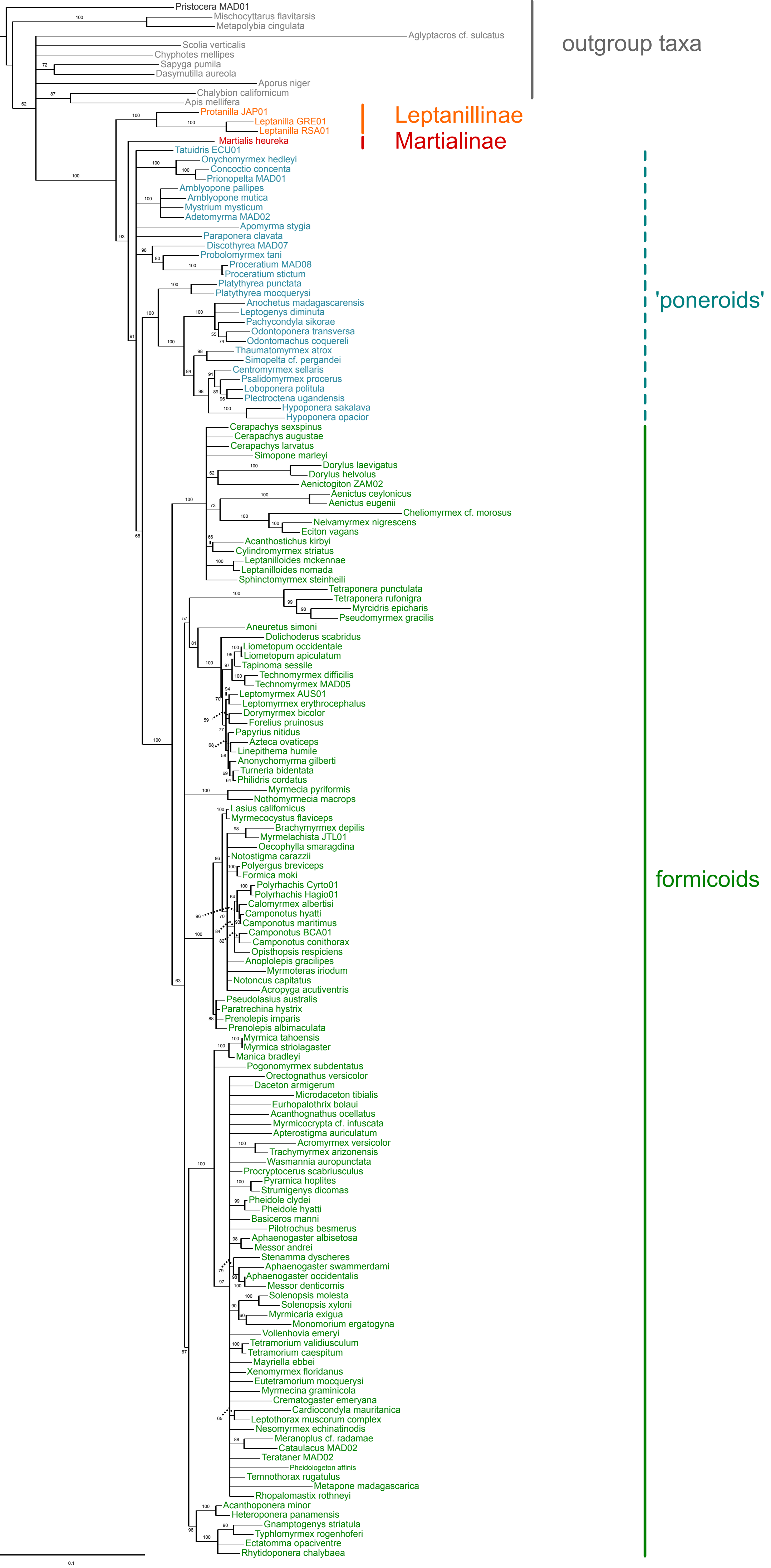


Figure S4 : Maximum likelihood (majority rule) inferred from the masked, partitioned data set with 5,000 bootstrap replicates (-f a; GTR + GAMMA, see method section). The tree was rooted with Pristocera.
