# Supplementary material for: Improved Phylogenetic Analyses Corroborate a Plausible Position of Martialis heureka in the Ant Tree of Life
Source: PLoS One. 2011 Jun 24;6(6):e21031. doi: 10.1371/journal.pone.0021031 (PMC3123331; doi:10.1371/journal.pone.0021031)
Supplement: Figure S5 — Bayesian-phylogram (majority rule consensus tree) inferred from the unmasked alignment (28,130,500 generations, samplefrequency 100, burn-in: 10% discarded). (PDF) [file pone.0021031.s005.pdf]

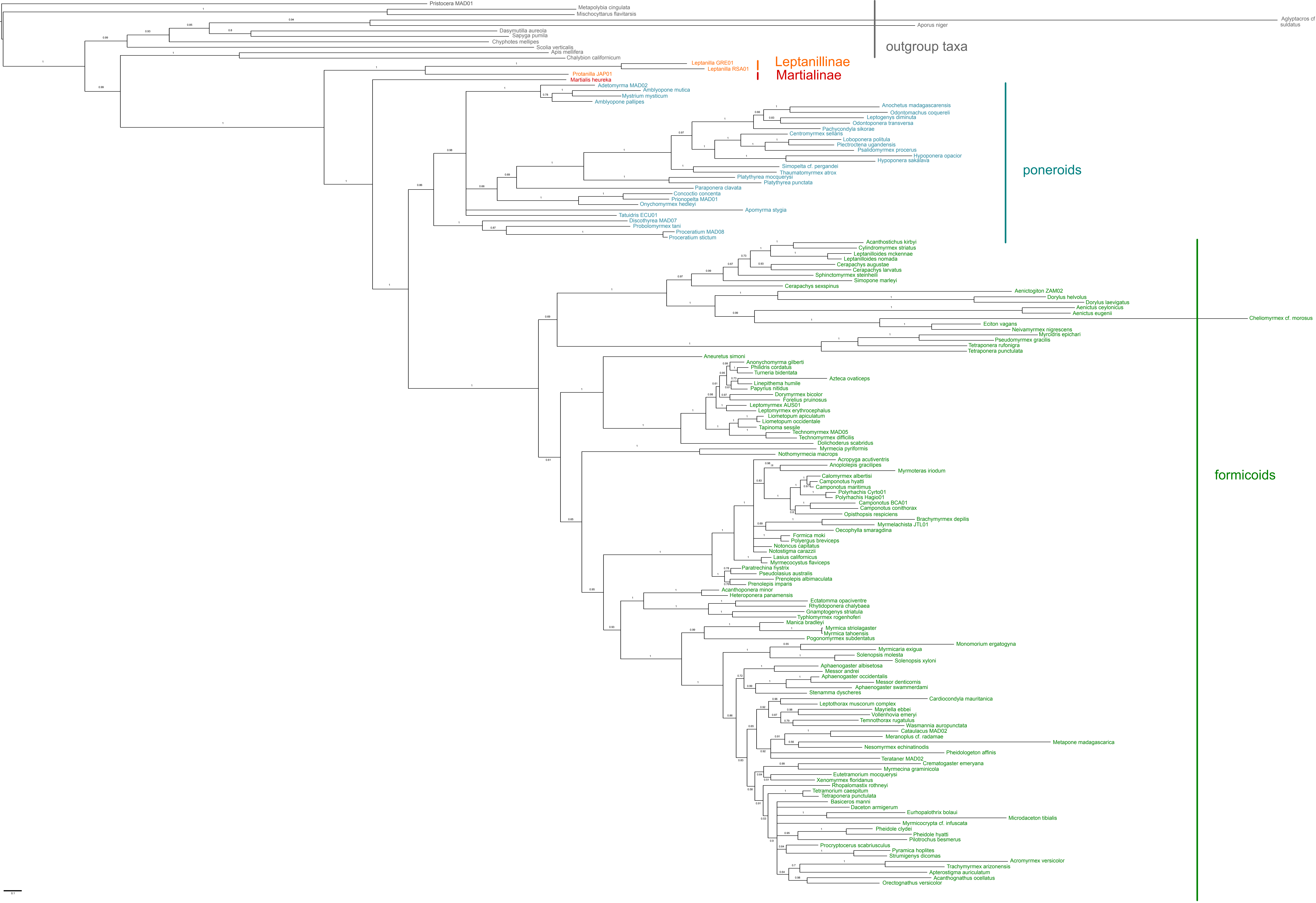

Figure S5: Bayesian tree (majority rule consensus) inferred from the unmasked, unpartitioned data set (GTR + GAMMA, 28,130,500 generations, sample frequency 100, burn-in (10%) discarded; see method section). The tree was rooted with Pristocera.
