# Supplementary figures and images for: Improved Phylogenetic Analyses Corroborate a Plausible Position of Martialis heureka in the Ant Tree of Life
Source: PLoS One. 2011 Jun 24;6(6):e21031. doi: 10.1371/journal.pone.0021031 (PMC3123331; doi:10.1371/journal.pone.0021031)

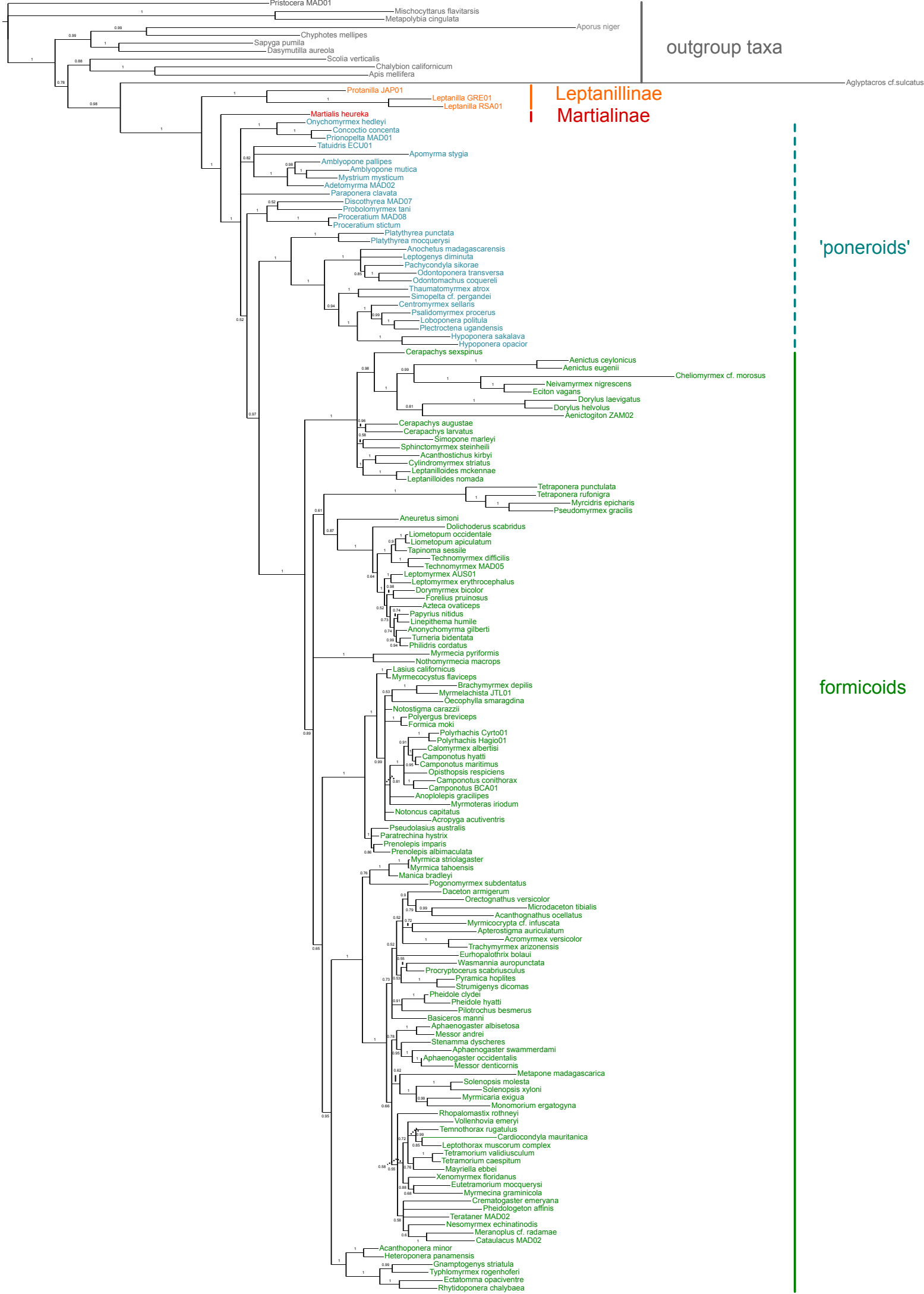

Supplement: Figure S6 — Bayesian-phylogram (majority rule consensus tree) inferred from the masked-unpartitioned approach (30 million generations, sample frequency 200, burn-in: 10% discarded). (PDF) [file pone.0021031.s006.pdf]
