# Supplementary material for: Improved Phylogenetic Analyses Corroborate a Plausible Position of Martialis heureka in the Ant Tree of Life
Source: PLoS One. 2011 Jun 24;6(6):e21031. doi: 10.1371/journal.pone.0021031 (PMC3123331; doi:10.1371/journal.pone.0021031)
Supplement: Figure S7 — Bayesian-phylogram (majority rule consensus tree) inferred from the masked-partitioned approach. (30 million generations, sample frequency 200, burn-in: 10% discarded). (PDF) [file pone.0021031.s007.pdf]

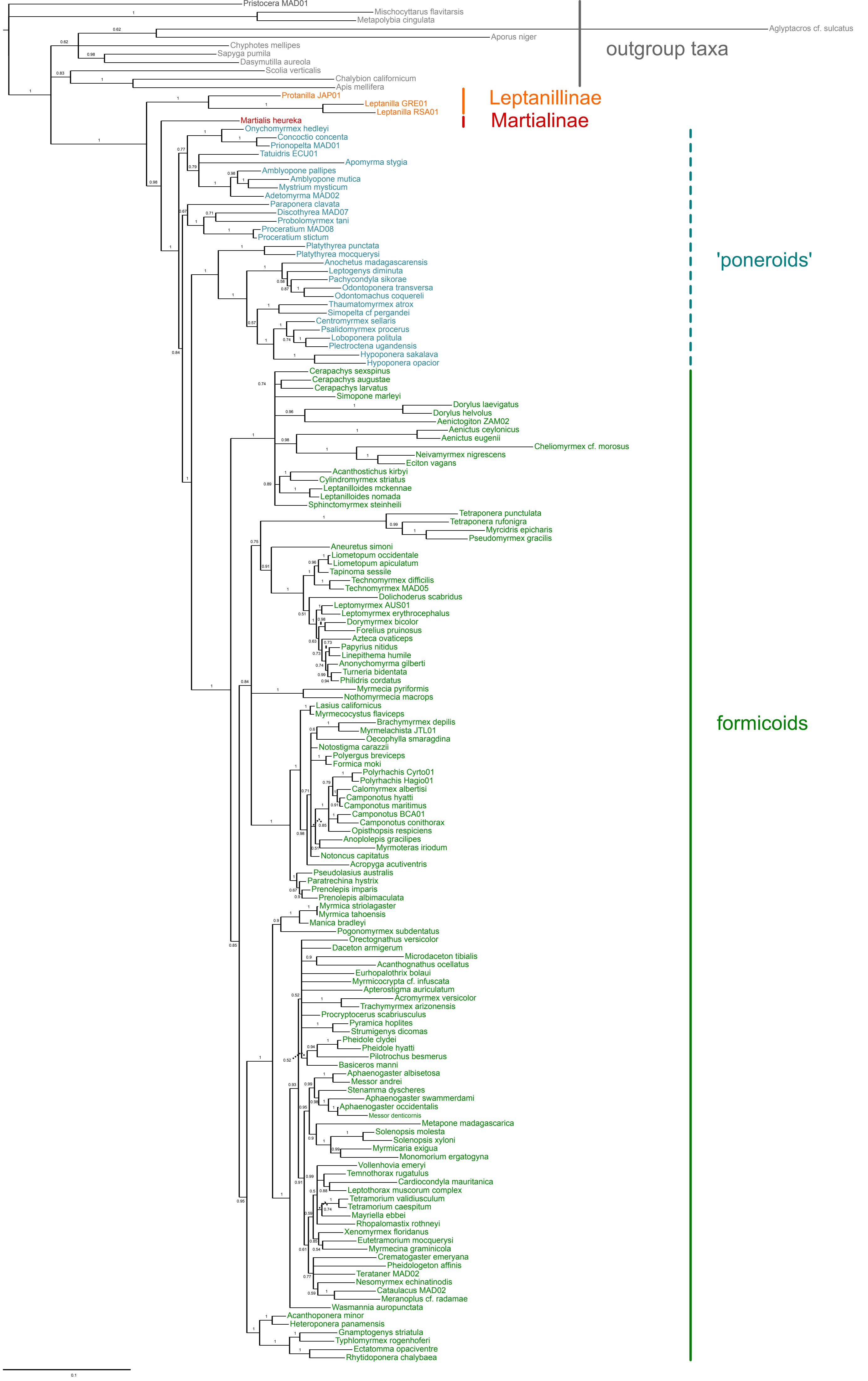

Figure S 7 : Bayesian tree (majority rule consensus) inferred from the masked, partitioned data set (GTR + GAMMA, 30 million geneRations, sample frequency 200, burn-in (10%) discarded; see method section). The tree was rooted with Pristocera.
